# Minimally invasive aortic valve replacement in morbidly obese patients: outcomes from a cohort study and pooled data analysis

**DOI:** 10.3389/fcvm.2025.1659991

**Published:** 2026-01-16

**Authors:** Lukman Amanov, Sadeq Ali-Hasan-Al-Saegh, Arian Arjomandi Rad, Antonia Annegret Jauken, Thanos Athanasiou, Jawad Salman, Fabio Ius, Stefan Rümke, Bastian Schmack, Arjang Ruhparwar, Alina Zubarevich, Alexander Weymann

**Affiliations:** 1 Department of Cardiothoracic, Transplantation and Vascular Surgery, Hannover Medical School, Hannover, Germany; 2 Department of Cardiothoracic Surgery, Oxford Heart Centre, John Radcliffe Hospital, Oxford University NHS Foundation Trust, Oxford, United Kingdom

**Keywords:** aortic valve replacement, minimally invasive cardiac surgery, ministernotomy, body mass index, obesity

## Abstract

**Background:**

In the context of cardiac surgery, morbid obesity poses several perioperative challenges. Some surgeons consider obesity a relative contraindication for minimally invasive aortic valve replacement (MIAVR) due to anatomical and technical complexities. Although MIAVR is increasingly used in standard-risk populations, evidence supporting its safety and efficacy in morbidly obese patients remains limited.

**Methods:**

This retrospective cohort consisted of 920 patients who underwent MIAVR via partial upper ministernotomy at a high-volume cardiac surgery center between 2010 and May 2025. Patients were categorized into three groups based on BMI: Class I obesity (BMI 30–35 kg/m^2^; *n* = 164), Class II–III obesity (BMI > 35 kg/m^2^; *n* = 54), and a non-obese control group (*n* = 702). Key clinical outcomes, echocardiographic parameters, postoperative complications, and long-term mortality rates were compared. Additionally, a pairwise meta-analysis was conducted, incorporating five studies to assess outcomes of MIAVR vs. conventional full sternotomy in obese individuals.

**Results:**

There were no significant differences in most of postoperative outcomes. However, higher rates of pneumothorax and arrhythmias were observed in Class II–III obesity. Multivariate regression did not identify obesity as an independent predictor of adverse outcomes. Meta-analysis confirmed comparable operative times and a trend toward shorter ICU stays and lower respiratory complications in the MIAVR group.

**Conclusion:**

This study argues that (i) obesity alone should not delay, deter, or preclude appropriate candidates from being referred for surgical aortic valve replacement, and (ii) partial upper ministernotomy should be considered the preferred access route in obese patients, as it consistently facilitates recovery without compromising safety.

## 1 Introduction

The rising prevalence of morbid obesity worldwide presents a significant challenge with major implications for public health (1). Over the past few decades, global obesity rates have risen sharply (1, 2). Current data show that approximately 40% of men are classified as overweight [body mass index (1) (BMI) 25–30 kg/m^2^], while 13% meet the criteria for obesity (BMI > 30 kg/m^2^) (1). Obesity significantly increases the risk of cardiovascular disease and is a well-established risk factor for aortic valve pathology, particularly calcific aortic stenosis (3). In the context of cardiac surgeries, morbid obesity presents multiple perioperative challenges, including impaired wound healing, higher infection risk, prolonged mechanical ventilation, and increased resource utilization (4–6).

Aortic valve disorders, such as stenosis and regurgitation, are common and pose substantial challenges in cardiovascular care (7). In the absence of treatment, severe symptomatic cases are associated with poor outcomes, with mortality rates ranging from 30% to 50% within the first year (8, 9). While conventional surgical aortic valve replacement (SAVR) continues to be the primary treatment option, many patients are deemed unsuitable for this approach because of elevated operative risk. Minimally invasive aortic valve replacement (MIAVR) has emerged as an alternative strategy aimed at reducing procedural trauma and improving patient recovery (10–13). Compared to full sternotomy, ministernotomy has demonstrated benefits including reduced bleeding, decreased postoperative pain, faster recovery, and shorter ICU and hospital stays, without compromising valve-related outcomes (10–12). Despite growing use of MIAVR in standard-risk populations, evidence specific to morbidly obese patients remains limited. The impact of obesity on clinical outcomes after transcatheter aortic valve implantation (TAVI) has been extensively studied in several clinical trials and meta-analyses (14, 15). Obesity is often regarded by some surgeons as a relative contraindication for MIAVR due to various challenges, including restricted mediastinal access, diminished visibility caused by elevation of the right hemidiaphragm from excess abdominal fat in the supine position, increased ventilation pressure requirements with a heightened risk of barotrauma, and complexities in managing venous drainage and arterial line pressures (16).

As the global burden of obesity continues to rise, it is critical to understand how minimally invasive techniques can be optimized for this complex patient subgroup. The first part of this study, designed as a retrospective cohort analysis, examines whether the anatomical and physiological characteristics of obese patients contribute to increased surgical complexity during MIAVR, and whether these factors negatively impact clinical outcomes compared to non-obese patients. Furthermore, we aim to assess whether these findings support a more cautious selection of obese patients for this surgical approach. The analysis was conducted at a high-volume cardiac surgical center in Germany, led by a highly experienced surgical team with expertise in perioperative management. Unlike previous studies, this study incorporates a more extensive patient population and a prolonged observation period extending to May 2025, enabling a thorough evaluation of long-term survival outcomes. To determine the specific influence of obesity on postoperative complications and mortality, we applied advanced statistical analyses, including multivariate regression models, to adjust for potential confounding variables. In addition, we conducted pooled meta-analyses to evaluate the safety and efficacy of MIAVR compared to conventional SAVR in obese patients.

## 2 Materials and methods

### 2.1 First part: retrospective cohort study

#### 2.1.1 Study population

Between January 2010 and April 2025, a total of 1200 MIAVR were performed at our center. After excluding cases with missing or incomplete data in our clinical documentation system (SAP software), 920 patients were included in the final analysis. Among them, 164 patients had a body mass index (BMI) of 30–35 kg/m^2^ (obesity class I), 54 patients had a BMI > 35 kg/m^2^ (obesity class II–III). Patients with class I obesity were categorized as Obesity Group A, while those with class II–III obesity were assigned to Obesity Group B. A total of 702 patients had a body mass index (BMI) below 30 kg/m^2^ and were classified as the non-obese group (Group C). All patients underwent MIAVR via partial upper ministernotomy. Perioperative and postoperative data were retrieved from our institutional database. The inclusion criteria encompassed all etiologies of aortic valve disease including degenerative, ischemic, rheumatic, and infective that were treated with MIAVR.

#### 2.1.2 Physical measurements

Height was measured without footwear using a standard wall-mounted stadiometer, while body weight was recorded with calibrated, physician-grade scales. All measurements were performed by trained staff to ensure consistency and accuracy. Body mass index (BMI) was calculated using the standard formula: BMI = weight (kg)/height^2^ (m^2^). According to widely accepted classifications, obesity was defined and categorized as follows: Class I (BMI 30 to <35 kg/m^2^), Class II (BMI 35 to <40 kg/m^2^), and Class III (BMI ≥ 40 kg/m^2^) (17).

#### 2.1.3 Ethical statement

As per local German regulations, ethical approval was not mandatory for this study due to its retrospective design and non-interventional nature.

#### 2.1.4 Surgical technique

All procedures were performed using a minimally invasive approach through a partial upper ministernotomy. The surgical technique, including cannulation strategy, aortic access, and valve implantation, was standardized and executed by an experienced surgical team. A 5–7 cm skin incision was made over the upper sternum, followed by a J-shaped or reverse T-shaped partial sternotomy extending into the third or fourth intercostal space. After opening the pericardium, which was suspended to optimize exposure, cardiopulmonary bypass (CPB) was established via direct cannulation of the ascending aorta and right atrium or via femoral vessels, depending on anatomical and technical considerations. Myocardial protection was achieved using cold antegrade crystalloid or blood cardioplegia, delivered directly into the coronary ostia following aortic cross-clamping. Once the heart was arrested, the ascending aorta was opened, and the diseased aortic valve was excised. A bioprosthetic or mechanical valve was then implanted using standard suturing techniques. Following valve replacement, the aortotomy was closed, and the heart was de-aired and reperfused. Protamine was administered to reverse systemic heparinization after the termination of CPB. Chest tubes were placed as needed, and the sternotomy was closed in layers.

#### 2.1.5 Follow-up and patient data collection

All patients were monitored for 30 days postoperatively, with survival status tracked through May 2025. Comprehensive data were collected, including demographic characteristics, echocardiographic findings, intraoperative details, and postoperative clinical outcomes. Postoperative complications assessed included mild and moderate-to-severe paravalvular leakage, structural valve deterioration, surgical explanation of failed valve, respiratory insufficiency, pneumothorax, arrhythmias (including new-onset atrial fibrillation (NOAF), atrioventricular (AV) block, and left bundle branch block (LBBB), need for permanent pacemaker implantation (PPM), right heart failure requiring extracorporeal membrane oxygenation (ECMO), Impella implantation, major bleeding requiring re-thoracotomy, thromboembolic events, ischemic stroke, seizures, delirium, intracranial hemorrhage, acute renal failure requiring dialysis, new myocardial infarction, cardiopulmonary resuscitation (CPR), sepsis, and late-onset endocarditis. Additional parameters recorded included the length of stay in the intensive care unit (ICU), intermediate care unit (IMC), and total hospital stay, as well as the number of transfused blood products.

#### 2.1.6 Outcome definitions

Early postoperative mortality was defined as death occurring during the index hospitalization or within 30 days of surgery, while late mortality referred to any death occurring beyond this 30-day period. Paravalvular leak (PVL) refers to any gap or channel between the anatomical annulus and the prosthetic valve, which leads to a regurgitation jet flowing between two heart chambers. Structural valve deterioration describes the intrinsic, acquired changes within the prosthesis, such as leaflet calcification, tears, fractures of the stent-frame, and damage to other components of the valve.

#### 2.1.7 Echocardiographic assessment

Transthoracic echocardiography (TTE) was routinely performed preoperatively and prior to hospital discharge. Key echocardiographic parameters collected included left ventricular ejection fraction (LVEF), severity grading of aortic valve regurgitation (AR) and aortic valve stenosis (AS), peak and mean transvalvular gradients, as well as the effective orifice area (EOA) of the aortic valve.

#### 2.1.8 Statistical analysis

All data analyses were carried out using IBM SPSS Statistics software (version 28.01.1). Continuous variables were summarized as mean values with their respective standard deviations (SD), while categorical data were reported as frequencies and percentages. To compare continuous variables between study groups, the Mann–Whitney *U* test was employed. Categorical data comparisons were conducted using the Chi-square test, and when expected cell frequencies were fewer than five, Fisher's exact test was applied instead. To examine associations between clinical variables and outcomes, multivariate logistic regression was employed for binary endpoints, and multivariate linear regression was used for continuous outcomes. Logistic regression was chosen to assess the likelihood of specific postoperative events (e.g., complications) based on multiple predictor variables, including BMI classification and other patient-related factors. The outputs of the logistic regression models were reported using the following metrics: for continuous variables, the unstandardized coefficient (B), its standard error (SE B), the standardized coefficient (*β*), the t-statistic (t), and the associated *p*-value; for categorical outcomes, the reported values included B, SE B, the Wald chi-square (*χ*²), and the corresponding *p*-value. Each regression model was analyzed separately. A two-sided *p*-value below 0.05 was considered statistically significant across all analyses.

### 2.2 Second part: pairwise meta-analysis

#### 2.2.1 Eligibility criteria and literature search

In accordance with the Cochrane Collaboration's methodology and following the standards outlined in the Preferred Reporting Items for Systematic Reviews and Meta-Analyses (PRISMA) guidelines, pairwise meta-analyses were conducted (18). The primary objective was to assess the safety, clinical effectiveness, and overall feasibility of MIAVR compared to conventional full sternotomy in morbidly obese patients with a body mass index (BMI) exceeding 30 kg/m^2^.

To ensure a structured and focused approach, the research question was developed using the PICOS (Population, Intervention, Comparator, Outcomes, Study design) framework. Studies were eligible for inclusion based on the following criteria:

- Population: Patients diagnosed with aortic valve disease of any etiology;
- Intervention: SAVR performed via partial upper ministernotomy;
- Comparator: Conventional SAVR performed via full sternotomy;
- Outcomes: Early and mid-term clinical outcomes, including perioperative complications, morbidity, and mortality;
- Study Design: Only original research articles were considered. Exclusion criteria comprised experimental studies, case reports, conference abstracts, editorials, letters to the editor, narrative reviews, and general commentaries.

A comprehensive literature search was conducted across multiple databases, including PubMed, PubMed Central, OVID Medline, the Cochrane Library. The search strategy incorporated a combination of relevant keywords and controlled vocabulary terms (e.g., MeSH), including: “aortic valve replacement,” “minimally invasive,” “ministernotomy,” “full sternotomy,” “obesity,” and “body mass index”. No restrictions were placed on the publication year. The final database search was completed in May 2025. Additionally, the reference lists of all included articles were manually reviewed to identify any additional studies that might not have been captured through the electronic database search.

#### 2.2.2 Data extraction

Two authors (SAHS and LA) independently evaluated all titles and abstracts retrieved from the literature search. Articles considered potentially relevant were then thoroughly examined in full by the same reviewers to verify eligibility and gather necessary information. In cases where disagreements arose during screening or data collection, these were discussed with senior authors (AZ and AW) until an agreement was reached.

#### 2.2.3 Outcome measures

For each study that met the inclusion criteria, key information was systematically collected, including the study design, country of origin, sample size, and median follow-up duration. In addition, patient demographic characteristics, types of aortic valve pathology, and preoperative risk profiles were recorded. Surgical details included the type of procedure performed, CPB time, ACC time, and total operative time. Clinical outcomes were categorized as follows: (1) Perioperative complications: acute renal failure, NOAF, re-exploration for bleeding, respiratory insufficiency, postoperative endocarditis, need for permanent pacemaker implantation, stroke, and wound dehiscence, (2) Resource utilization: duration of ICU stay and total hospital stay, (3) Mortality: early mortality (within 30 days), mid-term mortality, and late mortality (beyond follow-up period).

#### 2.2.4 Statistical analysis

Meta-analyses were carried out using RevMan (version 5.3.5), a software provided by The Cochrane Collaboration (Nordic Cochrane Centre, Copenhagen, Denmark). To account for potential heterogeneity among studies and to obtain broader and more robust estimates, a random-effects model based on the Mantel–Haenszel method was employed. Binary outcomes were evaluated by calculating odds ratios (ORs), while continuous data were summarized using weighted mean differences (MDs); both measures were reported with corresponding 95% confidence intervals (CIs). For included studies reporting medians and ranges without means and standard deviations, data were converted using the approach described by Hozo et al. (19). Statistical heterogeneity was assessed using the *I*^2^ statistic. Values of *I*^2^ ranging from 0%–25% were interpreted as negligible heterogeneity, 26%–50% as low, 51%–75% as moderate, and values exceeding 75% as high heterogeneity. A fixed-effects model was applied when *I*^2^ was less than 50%, while a random-effects model was preferred in cases where *I*^2^ exceeded 50%. The quality of the observational cohort studies was assessed using the Newcastle–Ottawa Scale (NOS).

## 3 Results

### 3.1 First part: retrospective cohort study

#### 3.1.1 Patient characteristics

As shown in Table 1, baseline patient characteristics were comparable across all three groups. The prevalence of most baseline comorbidities did not differ significantly between the groups. The mean age was 67.4 ± 11.6 years in the obesity class I group, 69.3 ± 12.6 years in the obesity class II–III group, and 72.2 ± 11.4 years in the non-obese group, with no statistically significant differences (*p* = 0.06). Similarly, the proportion of male patients was consistent across groups (*p* = 0.82). Most procedures were performed electively.

**Table 1:** Demographics and preoperative data.

Variables	Non-obese (*N* = 702)	Obesity Class I (*N* = 164)	Obesity Class II-III (*N* = 54)	*p*-value
Age	72.2 ± 11.4	67.4 ± 11.6	69.3 ± 12.6	0.06
Male Gender	377 (53.7%)	94 (57.3%)	31 (57.4%)	0.82
Elective operations	698 (99.4%)	161 (98.1%)	54 (100%)	0.19
Urgent operations	2 (0.2%)	3 (1.8%)	0 (0%)	
Emergency operations	0 (0%)	0 (0%)	0 (0%)	
Peripheral Artery Disease	250 (35.6%)	64 (39%)	21 (38.8%)	0.69
Renal impairment	153 (21.7%)	40 (24.3%)	17 (31.4%)	0.25
Haemodialysis	16 (2.2%)	4 (2.4%)	3 (5.5%)	0.32
Smoking history	164 (23.3%)	42 (25.6%)	15 (27.7%)	0.66
COPD	94 (13.3%)	24 (14.6%)	12 (22.2%)	0.16
Arterial hypertension	554 (78.9%)	142 (86.5%)	47 (87%)	0.03*
Hyperlipidemia	342 (48.7%)	108 (65%)	35 (64.8%)	0.001*
Recent Pneumonia	36 (5.1%)	10 (6%)	4 (7.4%)	0.71
Active endocarditis	5 (0.7%)	2 (1.2%)	0 (0%)	0.63
NIDDM	139 (19.8%)	63 (38.4%)	31 (57.1%)	0.001*
IDDM	79 (11.2%)	49 (29.8%)	26 (48.1%)	0.11
Coronary artery disease	384 (54.7%)	110 (67%)	32 (59.2%)	0.06
Recent myocardial infarction	117 (16.6%)	34 (20.7%)	5 (9.2%)	0.10
NYHA				
- I	413 (58.8%)	104 (63.4%)	34 (62.9%)	0.4
- II	151 (21.5%)	26 (15.8%)	7 (12.9%)	
- III	112 (15.9%)	27 (16.4%)	12 (22.2%)	
- IV	26 (3.7%)	7 (4.2%)	1 (1.8%)	
Preoperative stroke	61 (8.6%)	7 (4.2%)	3 (5.5%)	0.18
Neurological symptoms	86 (12.2%)	13 (7.9%)	3 (5.5%)	0.14
Pulmonary hypertension	163 (23.2%)	51 (31%)	12 (22.2%)	0.09
Atrial fibrillation	146 (20.7%)	36 (21.9%)	12 (22.2%)	0.92
Beta-blocker use	416 (59.2%)	101 (61.5%)	33 (61.1%)	0.81
Calcium channel blocker use	370 (52.7%)	73 (44.5%)	18 (33.3%)	0.007*
Amiodarone use	41 (5.8%)	7 (7.2%)	0 (0%)	0.14
Digoxin use	18 (2.5%)	3 (1.8%)	0 (0%)	0.43
Statin therapy	396 (56.4%)	94 (57.3%)	22 (40.7%)	0.08
Aortic valve stenosis	397 (56.6%)	79 (48.1%)	23 (42.5%)	0.02*
Aortic valve regurgitation	39 (5.5%)	14 (8.5%)	7 (12.9%)	0.02*
Combined pathologies	266 (37.8%)	71 (43.2%)	24 (44.4%)	0.38

NYHA, New York heart association; COPD, chronic obstructive pulmonary disease; IDDM, insulin dependent diabetes mellitus; NIDDM, non-insulin dependent diabetes mellitus.

* *p*-value significant.

#### 3.1.2 Procedural characteristics

Intraoperative details are summarized in Table 2. The average surgery duration, as well as CPB and ACC times, were notably longer in obese patients compared to those without obesity (*p* = 0.001). Procedures such as aortic root replacement, supracoronary ascending aorta replacement, tricuspid valve repair, Bentall and David procedures, mitral and tricuspid valve replacements or repairs, and Maze operations were carried out at comparable frequencies across both groups (*P* > 0.05).

**Table 2:** Intraoperative data.

Variables	Non-obese (*N* = 702)	Obesity Class I (*N* = 164)	Obesity Class II-III (*N* = 54)	*p*-value
Calcified aortic valve	585 (83.3%)	134 (81.7%)	38 (70.3%)	0.60
Bicuspid aortic valve	20 (2.8%)	16 (9.7%)	2 (3.7%)	0.03*
Rheumatic aortic valve	4 (0.5%)	2 (1.2%)	0 (0%)	0.54
Endocarditis	4 (0.5%)	0 (0%)	1 (1.8%)	0.27
Concomitant Aortic root replacement	7 (0.9%)	3 (1.8%)	3 (5.5%)	0.21
Concomitant Aortic annuloplasty	1 (0.1%)	2 (1.2%)	0 (0%)	0.85
Concomitant Supracoronary replacement of the ascending aorta	45 (6.4%)	11 (6.7%)	7 (12.9%)	0.18
Concomitant Bental procedure	3 (0.4%)	2 (1.2%)	0 (0%)	0.39
Concomitant David's procedure	4 (0.5%)	2 (1.2%)	3 (5.5%)	0.20
Concomitant Patent Foramen Ovale Closure	1 (0.1%)	1 (0.6%)	0 (0%)	0.58
Concomitant left atrial appendage closure	17 (2.4%)	5 (3%)	2 (3.7%)	0.78
Concomitant Maze Procedure	2 (0.2%)	0 (0%)	0 (0%)	0.75
Concomitant MVR	5 (0.7%)	5 (3%)	1 (1.8%)	0.42
Concomitant TVR	1 (0.1%)	0 (0%)	0 (0%)	0.86
Duration of surgery (Minutes)	141.6 ± 60	154.8 ± 66	152.4 ± 66.6	0.001*
Time on CPB (Minutes)	89.3 ± 44.2	112 ± 46.8	117.9 ± 57.9	0.001*
Duration of ACC (Minutes)	53.4 ± 28.4	69.7 ± 30.2	74.4 ± 34.8	0.001*

CPB, cardiopulmonary bypass; ACC, aortic cross clamp; MVR, mitral valve replacement; TVR, tricuspid valve repair or replacement. *p-value significant.

#### 3.1.3 Postoperative outcomes and complications

A summary of clinical outcomes and surgical complications across the three BMI groups is presented in Table 3. Overall, there were no statistically significant differences between the groups in the need for red blood cell, platelet, or fresh frozen plasma transfusions. Likewise, the average durations of ICU stay, IMC stay, and total hospital stay did not differ significantly among the groups (all *p*-values > 0.05).

**Table 3:** Post-operative outcomes and complications.

Variables	Non-obese (*N* = 702)	Obesity Class I (*N* = 164)	Obesity Class II-III (*N* = 54)	*p*-value
Erythrocyte transfusion	1.75 ± 2.0	1.5 ± 1.6	0.5 ± 0.3	0.15
FFP transfusion	0.23 ± 1.7	0.14 ± 1.2	0.15 ± 0.3	0.49
Platelet transfusion	0.24 ± 1.2	0.10 ± 1.0	0.03 ± 0.3	0.82
ICU stay (days)	2.4 ± 3.5	2.04 ± 2.8	2.22 ± 4.8	0.48
IMC stay (days)	1.03 ± 3.2	0.4 ± 1.8	1.17 ± 3.8	0.06
Hospital stay (days)	15.3 ± 8.1	15.1 ± 8.9	16 ± 7.5	0.76
Respiratory insufficiency	86 (12.2%)	23 (14%)	9 (16.6%)	0.56
Pneumothorax	58 (8.2%)	7 (4.2%)	11 (20.3%)	0.001*
Aortic valve re-operation	0 (0%)	0 (0%)	0 (0%)	1
Arrhythmia	161 (22.9%)	51 (31%)	20 (37.0%)	0.01*
New atrial fibrillation	96 (13.6%)	37 (22.5%)	14 (25.9%)	0.002*
AV Block II°	12 (1.7%)	3 (1.8%)	0 (0%)	0.61
AV Block III°	50 (7.1%)	13 (7.9%)	4 (7.4%)	0.93
LBBB	23 (3.2%)	7 (4.2%)	0 (0%)	0.39
Pacemaker implantation	50 (7.1%)	12 (7.3%)	4 (7.4%)	0.84
ECMO/right ventricular failure	5 (0.7%)	1 (0.6%)	0 (0%)	0.81
Impella	0 (0%)	1 (0.6%)	1 (1.8%)	0.05*
Re-thoracotomy	20 (2.8%)	8 (4.8%)	3 (5.5%)	0.28
Major Bleeding	37 (5.2%)	10 (6%)	3 (5.5%)	0.91
Vascular Injuries	9 (1.2%)	1 (0.6%)	1 (1.8%)	0.68
Renal failure with new onset dialysis	16 (2.2%)	5 (3.0%)	0 (0%)	0.42
Stroke	13 (1.8%)	3 (1.8%)	0 (0%)	0.60
Cerebral bleeding	2 (0.2%)	1 (0.6%)	0 (0%)	0.73
Seizure	10 (1.4%)	0 (0%)	0 (0%)	0.20
Delirium	73 (10.3%)	9 (5.4%)	6 (11.1%)	0.14
Neurologic deficits	28 (3.9%)	8 (4.8%)	3 (5.5%)	0.77
Thromboembolic events	15 (2.1%)	3 (1.8%)	2 (3.7%)	0.78
DVT	2 (0.2%)	0 (0%)	0 (0%)	0.73
Wound dehiscence	25 (3.5%)	6 (3.6%)	3 (5.5%)	0.75
- Deep	2 (0.2%)	0 (0%)	0 (0%)	
- Superficial	23 (3.3%)	6 (3.6%)	3 (5.5%)	
Sepsis	10 (1.4%)	5 (3.0%)	2 (3.7%)	0.22
Myocardial infarction	2 (0.2%)	0 (0%)	0 (0%)	0.73
Pacemaker implantation	50 (7.1%)	12 (7.3%)	5 (9.2%)	0.84
Mild Paravalvular leak	30 (4.2%)	13 (7.9%)	5 (9.2%)	0.65
Moderate-Severe Paravalvular leak	1 (0.1%)	0 (0%)	0 (0%)	0.86
SVD	1 (0.1%)	1 (0.6%)	0 (0%)	0.91
Surgical explantation of failed valve	0 (0%)	0 (0%)	0 (0%)	1
Late Endocarditis	5 (0.7%)	1 (0.6%)	0 (0%)	0.89
CPR	33 (4.7%)	6 (3.6%)	2 (3.7%)	0.81
In-hospital mortality	20 (2.8%)	4 (2.4%)	1 (1.8%)	0.53
30-day mortality	27 (3.8%)	4 (2.4%)	1 (1.8%)	0.44
Late mortality	39 (5.5%)	6 (3.6%)	3 (5.5%)	0.61

CPB, cardiopulmonary bypass; FFP, fresh frozen plasma; ECMO, extracorporeal membrane oxygenation; CPR, cardiopulmonary resuscitation; SVD, structural valve deterioration; LBBB, left bundle branch block; DVT, deep vein thrombosis; AV, atrioventricular block.

* *p*-value significant.

Respiratory complications such as respiratory insufficiency occurred at similar rates in all cases. However, the incidence of pneumothorax was significantly higher in patients with class II–III obesity (20.3%) compared to those with class I obesity (4.2%) and non-obese patients (8.2%) (*p* = 0.001). Additionally, both arrhythmias (*p* = 0.01) and NOAF (*p* = 0.002) were more frequently observed in obese patients. No significant differences were found regarding the occurrence of AV blocks, LBBB, the need for permanent pacemaker implantation and the occurrence of new onset myocardial infarction. Use of mechanical circulatory support such as ECMO was rare, and although Impella was used in only two patients, its use showed borderline statistical relevance (*p* = 0.05).

Re-thoracotomy was performed in 31 patients, with distribution across the groups as follows: 2.8% in the non-obese group, 4.8% in class I obesity, and 5.5% in class II–III obesity (*p* = 0.28). Major bleeding events and vascular injuries occurred at similar frequencies across all BMI categories. Neurological complications including stroke, seizures, delirium, cerebral bleeding and neurological deficits were also evenly distributed. Wound dehiscence was observed in 34 cases, with the following distribution: 3.5% in the non-obese group, 3.6% among patients with class I obesity and 5.5% in those with class II–III obesity (*p* = 0.75). Most wound dehiscences were superficial. Only 2 patients in the non-obese group experienced deep wound dehiscence. The incidence of postoperative renal failure requiring dialysis showed no significant variation by BMI. Valve-related complications within 30 days such as paravalvular leak, structural valve deterioration, and endocarditis were rare and showed no significant differences between the groups. A single case (0.1%) of severe paravalvular leak was reported in a non-obese patient. None of the patients required surgical removal of the implanted prosthetic valve.

Among the 35 in-hospital deaths out of 920 patients, the distribution across BMI groups was as follows: 2.8% in class I obesity, 2.4% in class II–III obesity, and 1.8% in the non-obese group (*p* = 0.53). Both 30-day and late mortality rates were comparable across all groups, with no statistically significant differences observe (*p*-values > 0.05).

#### 3.1.4 Echocardiographic assessments

Preoperative and postoperative echocardiographic data are summarized in Table 4. Following surgery, the mean LVEF remained similar across all groups, with no statistically significant difference observed (*p* = 0.98). A total of 39 patients (out of 920) exhibited moderate aortic regurgitation after MIAVR, distributed as follows: 3.4% in the non-obese group, 5.4% in class I obesity, and 11.1% in class II–III obesity (*p* = 0.06). Additionally, severe postoperative AR was documented in only three patients, all of whom were in the non-obese group (0.4%). No cases of moderate or severe aortic stenosis were identified after the procedure. Mean and peak transvalvular gradients were comparable between the groups (*P* > 0.05).

**Table 4:** Echocardiographic assessments .

Time	Variables	Non-obese (*N* = 702)	Obesity Class I (*N* = 164)	Obesity Class II-III (*N* = 54)	*p*-value
Preoperative	LVEF	56.9 ± 11.7	57 ± 10.6	57.5 ± 9	0.95
	Moderate AR	346 (49.2%)	50 (30.4%)	17 (31.4%)	0.03*
	Severe AR	17 (2.4%)	10 (6.0%)	4 (7.4%)	0.01*
	Mild AS	98 (13.9%)	25 (15.2%)	12 (22.2%)	0.36
	Moderate AS	438 (62.3%)	86 (52.4%)	22 (40.7%)	0.001*
	Severe AS	59 (8.4%)	18 (10.9%)	12 (22.2%)	0.001*
	Peak gradient *(mmHg)*	71.9 ± 17	71.8 ± 25.7	73.7 ± 29.4	0.83
	Mean gradient *(mmHg)*	44.8 ± 12.1	45.8 ± 16	46.8 ± 17.9	0.54
	EOA	1.7 ± 0.7	1.7 ± 1.5	1 ± 0.1	0.68
Postoperative	LVEF	54.3 ± 10.4	54.4 ± 10.2	54.5 ± 11	0.98
	Moderate AR	24 (3.4%)	9 (5.4%)	6 (11.1%)	0.06
	Severe AR	3 (0.4%)	0 (0%)	0 (0%)	0.62
	Mild AS	7 (0.9%)	3 (1.8%)	2 (3.7%)	0.22
	Moderate AS	0 (0%)	0 (0%)	0 (0%)	1
	Severe AS	0 (0%)	0 (0%)	0 (0%)	1
	Peak gradient *(mmHg)*	19.3 ± 4.1	20.6 ± 10.3	21.1 ± 8.9	0.42
	Mean gradient *(mmHg)*	10.1 ± 4.9	11.4 ± 5.7	11.4 ± 5.3	0.71

Pre-OP, preoperative; Post-OP, postoperative; LVEF, left ventricular ejection fraction; AR, aortic valve regurgitation; AS, aortic valve stenosis; EOA, effective orifice area.

*p-value significant.

#### 3.1.5 Regression model

In the multivariate logistic regression analysis (Supplementary Table S1), obesity was not identified as an independent predictor for hospitalization duration. Specifically, no statistically significant associations were observed with ICU stay (B = −0.22, SD = 0.22, *β* = 0.04, t = 0.96, *p* = 0.33), intermediate care stay (B = 0.14, SD = 0.22, *β* = 0.02, t = 0.67, *p* = 0.50), or total hospital stay (B = −0.32, SD = 0.60, *β* = –0.02, t = –0.54, *p* = 0.58). Obesity also did not emerge as an independent predictor of postoperative arrhythmias (B = 0.03, SD = 0.15, *β* = 0.05, t = 1, *p* = 0.81), and no other significant predictors for arrhythmia were identified. Similarly, the risk of new-onset atrial fibrillation was not significantly influenced by obesity (B = 0.01, SD = 0.17, *β* = 0.005, t = 1, *p* = 0.94). Furthermore, obesity was not associated with an increased likelihood of postoperative pneumothorax (B = −0.18, SD = 0.24, *β* = 0.59, t = 1, *p* = 0.44) or 30-day mortality (B = −0.36, SD = 0.60, *β* = 0.36, t = 1, *p* = 0.54).

### 3.2 Second part: pairwise meta-analysis

#### 3.2.1 Characteristics of included studies

**Figure 1:**
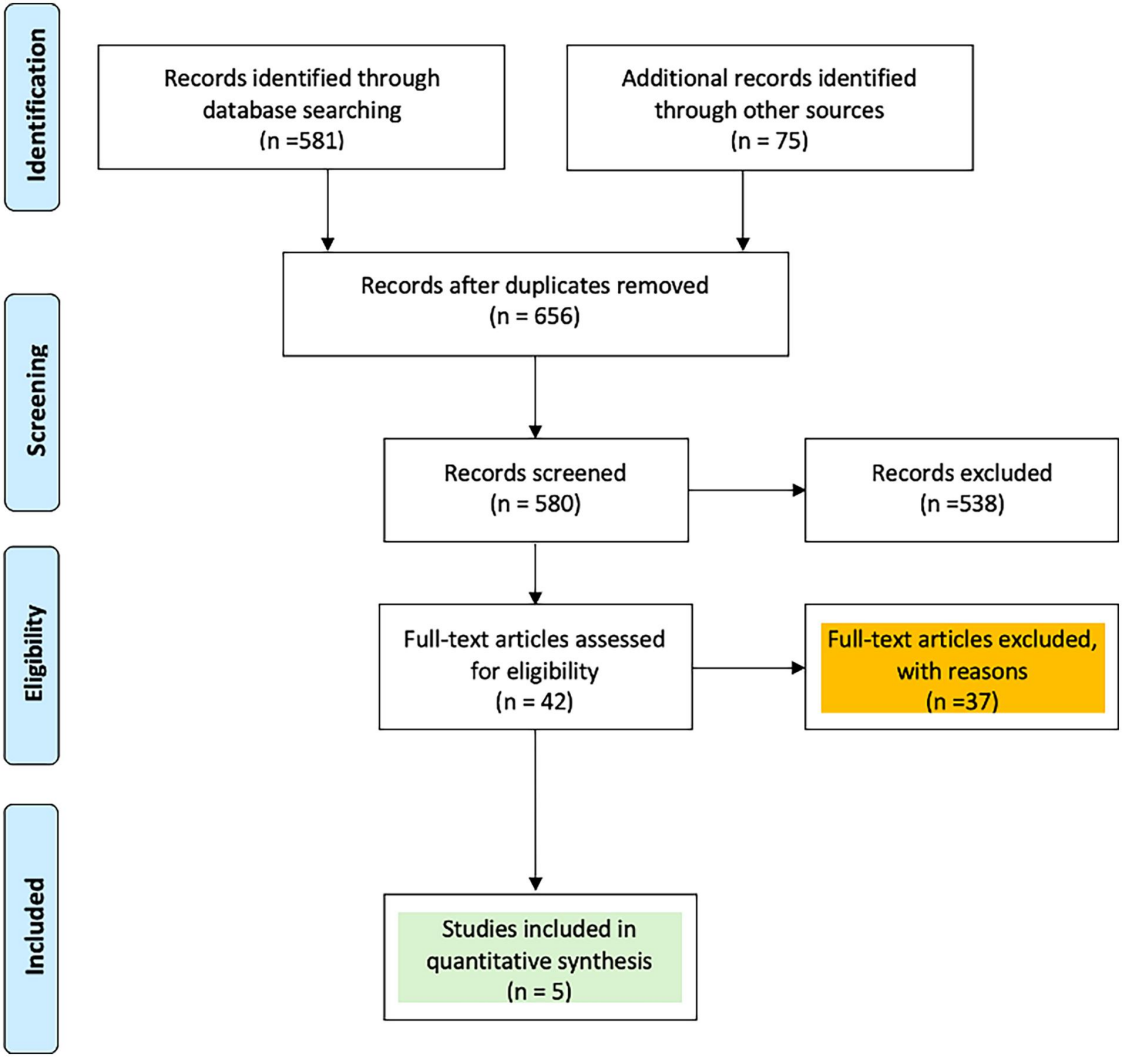
PRISMA flow chart of study selection.

The initial database search identified a total of 656 articles after duplicate removal. After a detailed full-text review of the remaining 42 articles, 37 were excluded for reasons such as lacking comparative data, containing irrelevant or repetitive information, or not adequately reporting the primary outcomes of interest (Figure 1). In the end, five studies (20–24) encompassing 1,050 patients were included in the final comparative pairwise meta-analysis. Among these, 460 morbidly obese patients underwent MIAVR via ministernotomy, while 590 patients received SAVR through full sternotomy (Supplementary Tables S2–S4).

**Figure 2:**
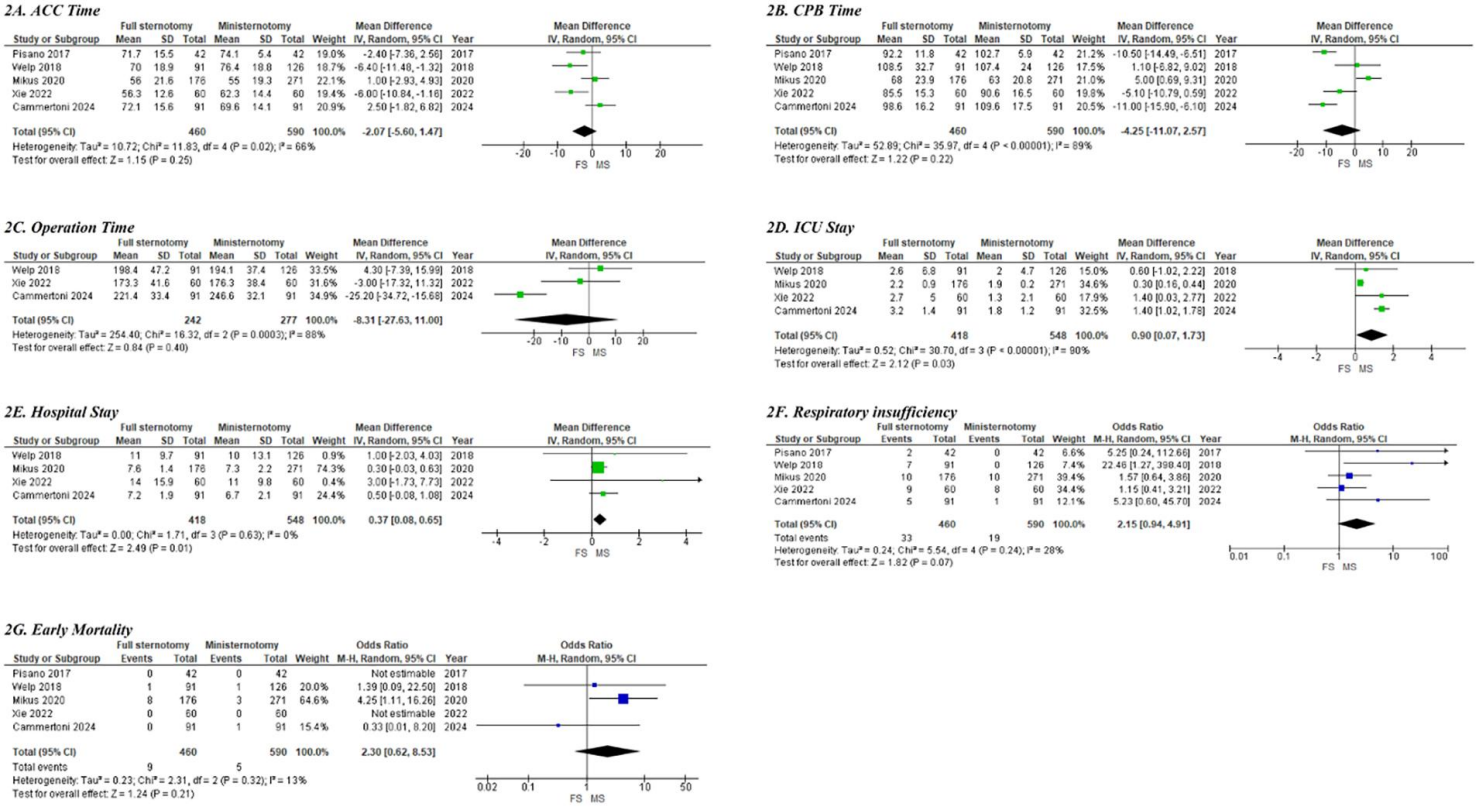
Forest plots of clinical outcomes and complications between conventional SAVR and MIAVR in morbidly obese cases; **(A)** ACC time; **(B)** CPB time; **(C)** operation time; **(D)** ICU stay; **(E)** hospital stay; **(F)** respiratory insufficiency; **(G)** early mortality.

The comparison between SAVR performed through full sternotomy and MIAVR via ministernotomy showed no statistically significant differences in ACC time (MD: −2.07, 95% CI: −5.60–1.47; *p* = 0.25; Figure 2A), CPB time (MD: −4.25, 95% CI: −11.07–2.57; *p* = 0.22; Figure 2B), or total operative duration (MD: −8.31, 95% CI: −27.63–11.0; *p* = 0.40; Figure 2C). However, pooled analysis demonstrated that, in obese patients, MIAVR via ministernotomy was associated with significantly shorter ICU stays (MD: 0.90, 95% CI: −0.07–1.73; *p* = 0.03; Figure 2D) and reduced overall hospital length of stay (MD: 0.37, 95% CI: 0.08–0.65; *p* = 0.01; Figure 2E).

The pooled analysis using a random-effects model demonstrated that MIAVR via ministernotomy did not confer any disadvantages compared to full sternotomy regarding the need for permanent pacemaker implantation (3% for conventional SAVR vs. 2.6% for MIAVR; OR: 1.13, 95% CI: 0.51–2.51; *p* = 0.76). Similarly, there were no significant differences between groups in the incidence of NOAF (26% vs. 27.3%; OR: 0.99, 95% CI: 0.72–1.36; *p* = 0.90) or stroke (2.6% vs. 1.15%; OR: 2.57, 95% CI: 0.62–10.74; *p* = 0.19) (Supplementary Figure S1). Moreover, rates of acute renal failure requiring dialysis, wound dehiscence, and the need for re-exploration due to major bleeding were statistically comparable between the two surgical approaches (Supplementary Figure S1).

Respiratory insufficiency was observed in 7.1% (33 of 460) of patients who underwent full sternotomy, compared with 3.2% (19 of 590) in the MIAVR via ministernotomy group. The pooled analysis indicated a trend toward a higher risk of postoperative respiratory insufficiency among obese patients treated with full sternotomy; however, this difference did not reach statistical significance (OR: 2.15; 95% CI: 0.94–4.91; *p* = 0.07; Figure 2F). Early mortality did not differ significantly between the two surgical approaches in obese patients (1.9% in the conventional SAVR group vs. 0.8% in the MIAVR group; OR: 2.30; 95% CI: 0.62–8.53; *p* = 0.21; Figure 2G). The quality of the observational cohort studies, as assessed using the Newcastle–Ottawa scale, is presented in Supplementary Table S5.

## 4 Discussion

The present study examined the impact of obesity on early outcomes after SAVR in a contemporary single-center cohort and, in parallel, performed a focused meta-analysis of obese patients undergoing SAVR through a full sternotomy vs. an upper mini-sternotomy. Three key messages emerge. First, obesity *per se* lengthened CPB and ACC times but did not translate into excess mortality or resource consumption. Second, extreme obesity (BMI ≥ 35 kg/m^2^) was accompanied by higher crude rates of atrial arrhythmia and pneumothorax, yet multivariable modeling failed to confirm obesity as an independent predictor of any early complication. Third, across five contemporary observational studies involving >1,100 obese patients, ministernotomy was associated with shorter intensive care and hospital stay and lower respiratory morbidity than full sternotomy, with comparable mortality. These observations have important implications for operative planning in the growing population of obese patients referred for SAVR.

### 4.1 Obesity, operative efficiency, and the “paradox” of unchanged mortality

The heaviest patients in our cohort were younger (mean age 69 ± 13 years in class II–III obesity vs. 72 ± 11 years in non-obese) yet accumulated more cardiovascular risk factors—arterial hypertension (87% vs. 79%) and hyperlipidaemia (65% vs. 49%) stood out. workflows that compensate for longer exposure and cannulation phases. In our 920-patient cohort, both CPB (89 ± 44 min vs. 118 ± 58 min) and ACC times (53 ± 28 min vs. 74 ± 35 min) increased steadily from non-obese to class II–III obesity (*p* < 0.001). Similar prolongation has been reported previously and is thought to reflect technical challenges in exposure, cannulation, and hemostasis in the deep thoracic field of obese patients. The absence of a parallel increase in total operative time reflects streamlined anaesthetic and perfusion Despite this, 30-day (3.8%) and in-hospital (2.9%) mortality were low and did not differ by BMI class, echoing the well-described “obesity paradox” (25). In a nationwide analysis of >90,000 cardiac surgical procedures, Mariscalco and colleagues found the nadir of risk in the overweight range, with no excess mortality until BMI exceeded 40 kg/m^2^ (26). More recent registry work confirmed that 30-day survival is at least equivalent, and transfusion requirements lower, in obese compared with normal-weight patients (27).

Several mechanisms have been proposed to account for this paradox. Obese patients have greater metabolic reserve, a blunted catecholamine response, and are often younger at surgery. Conversely, underweight patients may represent a frailer, cachectic subgroup with sarcopenia and systemic inflammation (28). The neutral association we observed after rigorous adjustment for 14 comorbid variables supports the notion that BMI alone should not preclude referral for SAVR.

### 4.2 Arrhythmia, pulmonary events, and the limits of BMI as a risk marker

Crude rates of NOAF increased from 13.7% in non-obese to 25.9% in class II–III obesity (*p* = 0.002). However, obesity did not remain an independent predictor after correction for age, hypertension and valvular pathology. Large administrative datasets likewise suggest that postoperative AF is driven mainly by pre-existing atrial remodeling, left-atrial hypertension, and systemic inflammation rather than BMI *per se* (29). We also observed a higher incidence of pneumothorax in the most obese stratum, which probably reflects both longer operative time and the more aggressive use of pleural drains to optimize ventilation in these patients. The occurrence of pneumothorax may be related to longer operative time, which could be due to intraoperative technical challenges or unintended lung injury, resulting in a more complex and extended procedure. On the other hand, longer operative times may require more lung recruitment maneuver or manipulation, which could also lead to the development of pneumothorax. Importantly, neither event translated into prolonged ICU or ward stay after multivariate adjustment (*β* = –0.04 days, *p* = 0.33 for BMI and ICU stay). These findings reaffirm that routine postoperative pathways can be safely applied across the BMI spectrum when meticulous intra-operative lung protection and rhythm surveillance protocols are followed.

### 4.3 Ministernotomy in obese patients—less morbidity without survival penalty

Our meta-analysis pooled five observational cohorts with a mean BMI of 32 kg/m^2^. Across studies, ministernotomy reduced ICU stay by a weighted mean of 0.6 days and hospital stay by 1.1 days and halved the odds of respiratory insufficiency (pooled OR 0.48). Early mortality was rare and statistically indistinguishable (1.1% conventional SAVR vs. 0.8% MIAVR). These results align with the most recent comprehensive meta-analysis of 48 studies by Khalid et al., who reported lower operative mortality and shorter length of stay with MIAVR (10). They also mirror the randomized UK Mini-Stern trial, in which ministernotomy failed to shorten transfusion requirements but was associated with faster functional recovery and better early quality of life (30).

The benefits of a smaller access appear particularly relevant in obese patients, who are prone to impaired diaphragmatic excursion and atelectasis. Although CPB and ACC times were slightly longer in ministernotomy cohorts (e.g., +11 min CPB in Liu et al.), this did not offset gains in postoperative recovery and did not influence mortality—a trade-off confirmed in propensity-matched series from high-volume centers (31). Combined with the consistent reduction in sternal-wound complications reported elsewhere (32), our data support ministernotomy as the default access in obese patients requiring isolated AVR, provided adequate exposure can be guaranteed.

### 4.4 Clinical implications

Taken together, the present findings suggest that (1) obesity alone should not deter referral for SAVR; (2) peri-operative teams should anticipate longer CPB/ACC times but not necessarily longer hospitalization; and (3) whenever technically feasible, an upper ministernotomy affords measurable advantages in resource use and pulmonary morbidity without compromising survival in obese patients. These observations are particularly pertinent as TAVI volumes rise, because severely obese patients derive no clear survival advantage from TAVI over SAVR and remain at risk of device malposition and vascular injury (21, 33).

### 4.5 Study limitations

Our institutional analysis is retrospective, and although comprehensive covariate adjustment was performed, residual confounding cannot be excluded. The relatively small number of class III (BMI≥40 kg/m^2^) patients limits inference at the extreme of the spectrum. Echocardiographic follow-up was restricted to the index hospitalization or within 30 days of surgery; longer-term valve hemodynamics and functional status were beyond the scope of the present work. Another limitation of this study is the lack of data on prosthesis-patient mismatch. Given that obese patients may have smaller annuli relative to body size, prosthesis-patient mismatch could represent an important confounding factor that influences long-term hemodynamic and clinical outcomes. The absence of this data limits the comprehensive interpretation of our findings. We acknowledge this limitation and plan to address it in future research to provide more detailed insights. The current study focused solely on ministernotomy as the minimally invasive approach. Future studies will aim to include other minimally invasive techniques, such as right anterior thoracotomy (RAT) and other endoscopic routes for aortic valve replacement, to provide a more comprehensive evaluation of available options. With respect to the meta-analysis, all included studies were observational and at moderate risk of bias (Newcastle–Ottawa score 7–8); heterogeneity in valve type, surgeon experience, and peri-operative protocols may influence pooled estimates. Nonetheless, consistency of direction and magnitude across studies lends credibility to the main conclusions.

## 5 Conclusions

In the modern surgical era, obesity no longer constitutes a prohibitive risk for patients undergoing isolated surgical aortic valve replacement. In our 920-patient, single-center cohort, increasing BMI lengthened CPB and ACC times but did not translate into higher early mortality, longer hospitalization, or greater resource consumption once comorbid burden was accounted for. Complementing these institutional findings, a focused meta-analysis of five contemporary studies involving 1,050 obese patients demonstrated that an upper ministernotomy yields clear peri-operative advantages—approximately half a day less in the ICU and on the ward, and a 45% reduction in postoperative respiratory insufficiency—without any survival penalty. Taken together, these data argue that (i) obesity alone should never delay, deter, or divert appropriate candidates from referral for SAVR, and (ii) ministernotomy should be considered the default access in obese patients when institutional expertise permits, as it reliably improves recovery while preserving safety. As the prevalence of severe obesity continues to rise, multicenter randomized trials focused on class III obesity, long-term valve durability, and patient-reported outcomes will be essential to refine guidelines and optimize care for this growing population.

## Data Availability

The original contributions presented in the study are included in the article/supplementary material, further inquiries can be directed to the corresponding author/s.

## Supplementary material

Supplementary Figure S1 Forest plots of clinical outcomes and complications in both groups.

Supplementary Table S1 Multivariate logistic regression analysis.

Supplementary Table S2 Characteristics of included articles for the second part of the study.

Supplementary Table S3 Postoperative clinical outcomes and complications in both groups.

Supplementary Table S4 Postoperative clinical outcomes and complications in both groups (continued).

Supplementary Table S5 Quality of the observational cohort studies, based on the Newcastle–Ottawa scale.
