# Supplementary figures and images for: Minimally invasive aortic valve replacement in morbidly obese patients: outcomes from a cohort study and pooled data analysis
Source: Front Cardiovasc Med. 2026 Jan 16;12:1659991. doi: 10.3389/fcvm.2025.1659991 (PMC12856294; doi:10.3389/fcvm.2025.1659991)

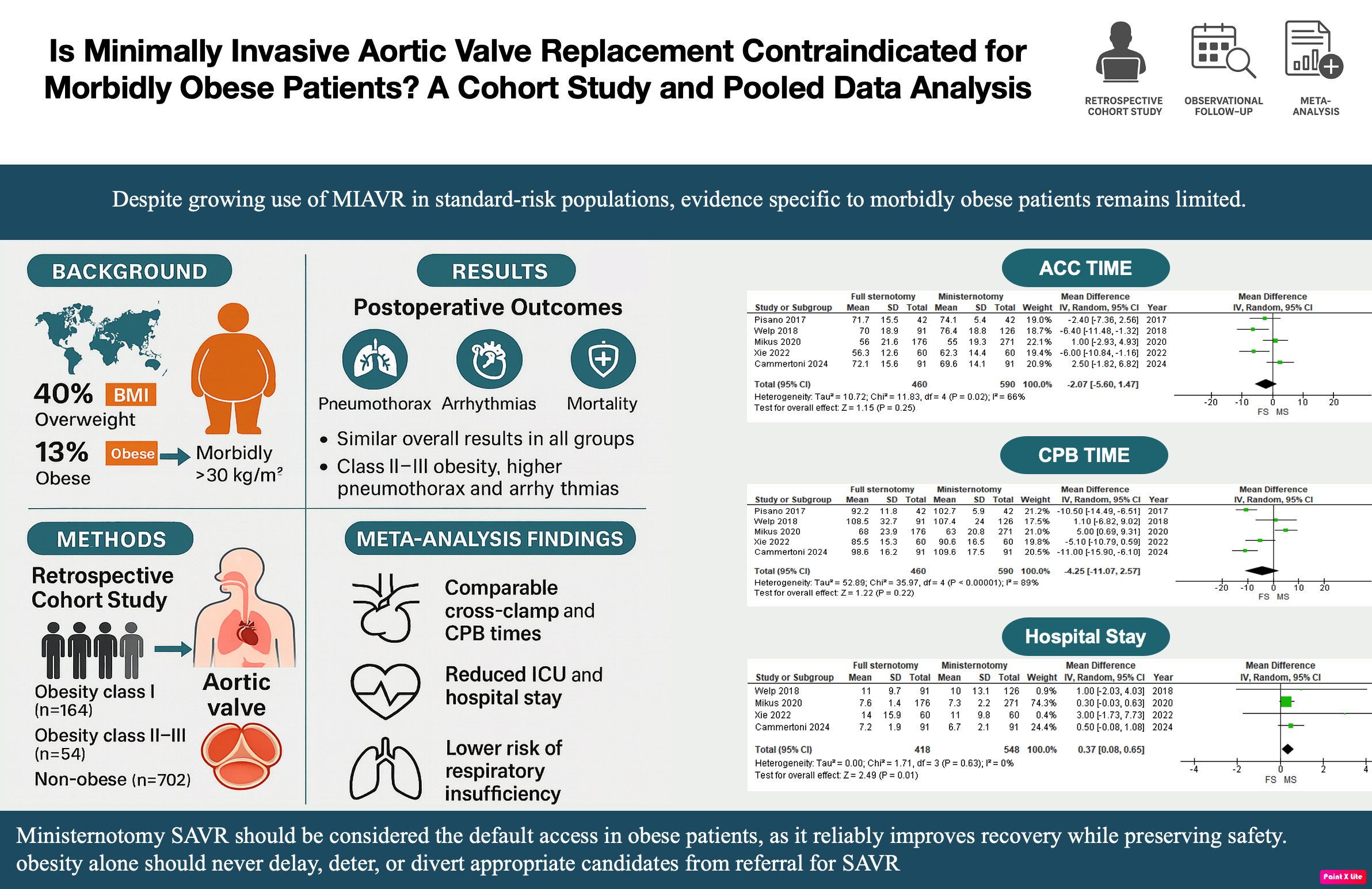

Supplement: Supplementary Figure S1 — Forest plots of clinical outcomes and complications in both groups. [file Image1.png]
