# Supplementary material for: Minimally invasive aortic valve replacement in morbidly obese patients: outcomes from a cohort study and pooled data analysis
Source: Front Cardiovasc Med. 2026 Jan 16;12:1659991. doi: 10.3389/fcvm.2025.1659991 (PMC12856294; doi:10.3389/fcvm.2025.1659991)

SUPPLEMENTARY DATA
Supplementary Table S1.

Continuous Quantitative Data											
ICU stay (days)						IMC stay (days)					
Variables	***Unstandardized.Coefficients***		***Standardized.Coefficients***		***Sig.***	*Variables*	***Unstandardized.Coefficients***		***Standardized.Coefficients***		***Sig.***
	B	***Std. Error***	***Beta***	***t***			***B***	***Std. Error***	***Beta***	***t***	
Obesity	-0.22	0.22	-0.04	-0.96	0.33	*Obesity*	0.14	0.22	0.02	0.67	0.50
NIDDM	-0.12	0.31	-0.01	-0.40	0.68	*NIDDM*	-0.01	0.29	-0.002	-0.04	0.96
Arterial HTN	-0.10	0.28	-0.01	-0.36	0.71	*Arterial HTN*	0.15	0.28	0.02	0.55	0.58
HLP	-0.13	0.24	-0.02	-0.56	0.57	*HLP*	-0.14	0.23	-0.02	-0.63	0.52
CCB	0.04	0.24	0.007	0.18	0.85	*CCB*	0.72	0.23	0.11	3.03	0.003
Pure AS	-0.61	0.34	-0.09	-1.78	0.07	*Pure AS*	0.32	0.33	0.05	0.96	0.33
Pure AR	-0.65	0.60	-0.05	-1.08	0.27	*Pure AR*	-0.35	0.58	-0.02	-0.61	0.53
Bicuspid valve	-0.39	0.60	-0.02	-0.64	0.51	*Bicuspid valve*	0.92	0.58	0.06	1.58	0.11
Moderate AR	-0.16	0.49	-0.02	-0.32	0.74	*Moderate AR*	0.61	0.48	0.08	1.26	0.20
Severe AR	-0.37	0.78	-0.02	-0.48	0.63	*Severe AR*	0.01	0.76	0.001	0.01	0.98
Moderate AS	-0.29	0.34	-0.04	-0.86	0.39	*Moderate AS*	0.07	0.32	0.01	0.21	0.83
Severe AS	-0.47	0.46	-0.04	-1.02	0.30	*Severe AS*	0.34	0.44	0.03	0.77	0.44
**Hospital stay (days)**						**Discrete Quantitative Data**					
Variables	***Unstandardized.Coefficients***		***Standardized.Coefficients***		***Sig.***	**Arhythmie**					
	B	***Std. Error***	***Beta***	***t***		***Variables***	***B***	***S.E.***	***Wald***	***df***	***Sig.***
Obesity	-0.32	0.60	-0.02	-0.54	0.58	*Obesity*	0.03	0.15	0.05	1	0.81
NIDDM	-1.01	0.82	-0.05	-1.24	0.21	*NIDDM*	0.09	0.23	0.16	1	0.68

Arterial HTN	0.68	0.76	0.03	0.89	0.37	*Arterial HTN*	-0.22	0.21	1.04	1	0.30
HLP	0.70	0.64	0.04	1.09	0.27	*HLP*	-0.01	0.18	0.004	1	0.94
CCB	0.05	0.65	0.003	0.07	0.93	*CCB*	0.01	0.18	0.009	1	0.92
Pure AS	0.47	0.91	0.02	0.52	0.59	*Pure AS*	-0.36	0.27	1.74	1	0.18
Pure AR	-3.83	1.59	-0.11	-2.41	0.01	*Pure AR*	-0.43	0.43	0.99	1	0.31
Bicuspid valve	1.17	1.60	0.02	0.73	0.46	*Bicuspid valve*	-0.38	0.43	0.78	1	0.37
Moderate AR	1.70	1.31	0.08	1.29	0.19	*Moderate AR*	-0.04	0.38	0.01	1	0.91
Severe AR	0.10	2.06	0.002	0.05	0.96	*Severe AR*	0.40	0.55	0.53	1	0.46
Moderate AS	-2.12	0.90	-0.12	-2.35	0.01	*Moderate AS*	0.09	0.23	0.14	1	0.70
Severe AS	-1.14	1.22	-0.04	-0.93	0.34	*Severe AS*	0.40	0.32	1.58	1	0.20
			**New-onset atrial fibrillation**						**Pneumothorax**		
Variables	***B***	***S.E.***	***Wald***	***df***	***Sig.***	***Variables***	***B***	***S.E.***	***Wald***	***df***	***Sig.***
Obesity	0.01	0.17	0.005	1	0.94	*Obesity*	-0.18	0.24	0.59	1	0.44
NIDDM	0.006	0.29	0.000	1	0.98	*NIDDM*	0.35	0.35	0.96	1	0.32
Arterial HTN	-0.08	0.26	0.10	1	0.74	*Arterial HTN*	-0.25	0.34	0.53	1	0.46
HLP	-0.12	0.21	0.31	1	0.57	*HLP*	0.29	0.30	0.99	1	0.32
CCB	-0.09	0.22	0.18	1	0.66	*CCB*	-0.18	0.28	0.39	1	0.53
Pure AS	-0.53	0.37	2.05	1	0.15	*Pure AS*	-1.08	0.56	3.65	1	0.05
Pure AR	-0.07	0.46	0.02	1	0.87	*Pure AR*	-1.12	0.76	2.13	1	0.14
Bicuspid valve	-0.04	0.45	0.008	1	0.93	*Bicuspid valve*	-0.34	0.72	0.22	1	0.63
Moderate AR	0.27	0.47	0.33	1	0.56	*Moderate AR*	-1.23	0.73	2.85	1	0.09
Severe AR	0.52	0.62	0.71	1	0.39	*Severe AR*	-0.01	0.84	0.000	1	0.98
Moderate AS	0.39	0.27	2.07	1	0.149	*Moderate AS*	-0.42	0.32	1.77	1	0.18

Severe AS	0.57	0.37	2.34	1	0.12	*Severe AS*	-0.75	0.54	1.90	1	0.16
			**Impella**						**30-day mortality**		
Variables	***B***	***S.E.***	***Wald***	***df***	***Sig.***	***Variables***	***B***	***S.E.***	***Wald***	***df***	***Sig.***
Obesity	14.06	904.38	0.0	1	0.98	*Obesity*	-0.36	0.60	0.36	1	0.54
NIDDM	-7.36	3790.30	0.0	1	0.99	*NIDDM*	1.04	0.57	3.34	1	0.06
Arterial HTN	6.28	4204.19	0.0	1	0.99	*Arterial HTN*	-0.73	0.60	1.46	1	0.22
HLP	-13.60	1256.24	0.0	1	0.99	*HLP*	1.35	0.63	4.48	1	0.34
CCB	-1.75	3192.86	0.0	1	1.0	*CCB*	-1.47	0.59	6.13	1	0.01
Pure AS	7.46	6306.47	0.0	1	0.99	*Pure AS*	-0.63	0.72	0.75	1	0.38
Pure AR	-7.28	7359.97	0.0	1	0.99	*Pure AR*	-17.23	4525.95	0.0	1	0.99
Bicuspid valve	-2.09	4663.67	0.0	1	1.0	*Bicuspid valve*	2.28	1.38	2.71	1	0.09
Moderate AR	10.28	11458.91	0.0	1	0.99	*Moderate AR*	-0.81	1.24	0.42	1	0.51
Severe AR	3.85	8590.96	0.0	1	1.0	*Severe AR*	-14.34	5382.39	0.0	1	0.99
Moderate AS	11.43	3859.11	0.0	1	0.99	*Moderate AS*	0.15	0.79	0.03	1	0.84
Severe AS	3.57	21242.65	0.0	1	1.0	*Severe AS*	-16.83	3886.49	0.0	1	0.99

Supplementary Table S2.

Author	Year	Intervention	No. of Patients	Mean BMI (kg/m2)	Male (n)	Mean age (year)	HTN (n)	DM (n)	EuroSCORE II	EF (%)	Study type
Cammertoni et al.	2024	FS	91	32.6 ± 1.2	54	69.2±13.1	75	22	ND	60.1±5.5	Cohort
		MS	91	32.9±1.3	55	68.6±14.5	76	18	ND	61.3±6.2	
Xie et al.	2022	FS	60	35.9 ± 4.5	32	60.1 ± 10.9	46	6	7.0 ± 6.1	59 ± 9.9	Cohort
		MS	60	34.9 ± 5.1	28	60.6 ± 11.3	43	5	7.0 ± 6.1	60.3 ± 8.3	
Mikus et al.	2020	FS	176	32.2 ± 2.2	82	72.7 ± 8.2	134	55	7.01 ± 1.5	59.7 ± 6.7	Cohort
		MS	271	32.8 ± 2.5	145	74.3 ± 7.5	203	64	6.7 ± 2.2	60 ± 7.5	
Pisano et al.	2017	FS	42	29.5 ± 8.5	21	76.5 ± 61.1	37	3	6.4 ± 3.8	--	Cohort
		MS	42	30.4 ± 6.1	15	76 ± 33.1	30	9	5.0 ± 4.6	--	
Welp et al.	2018	FS	91	33.11 ± 3.04	51	70.02 ± 10.51	87	35	7.96 ± 7.48	58.33 ± 10.82	Cohort
		MS	126	32.56 ± 3.09	69	69.79 ± 10.38	106	36	8.58 ± 8.78	55.16 ± 14.90	

Supplementary Table S3.

Author	Intervention	Renal Failure	AF	Re-Expl.	Respiratory insufficiency	Endocarditis	Need for PPM	Stroke	Wound dehiscence	ICU Stay	Hospital Stay
Cammertoni et al.	FS	0 (0%)	23 (25.3%)	5 (5.5%)	5 (5.5%)	ND	2 (2.2%)	1 (1.1%)	5 (5.5%)	3.2±1.4	7.2±1.9
	MS	0 (0%)	18 (19.8%)	3 (3.3%)	1 (1.1%)	ND	4 (4.4%)	1 (1.1%)	2 (2.2%)	1.8±1.2	6.7±2.1
Xie et al.	FS	2 (3.3%)	ND	0 (0%)	9 (15%)	ND	ND	ND	0 (0%)	2.7±5	14±15.9
	MS	3 (5%)	ND	1 (1.7%)	8 (13.3)	ND	ND	ND	1 (1.7%)	1.3±2.1	11±9.8
Mikus et al.	FS	8 (4.5%)	69 (39.2%)	ND	10 (5.7%)	ND	6 (3.4%)	ND	4 (2.3%)	2.2±0.9	7.6±1.4
	MS	4 (1.4%)	117 (43.2%)	ND	10 (3.7%)	ND	7 (2.6%)	ND	2 (0.7%)	1.9±0.2	7.3±2.2
Pisano et al.	FS	1 (2.1%)	10 (23.8%)	0 (0%)	2 (4%)	ND	2 (4.8%)	0 (0%)	0 (0%)	Prolonged stay 12 (30%)	Prolonged stays 33 (80%)
	MS	0 (0%)	6 (18.2%)	0 (0%)	0 (0%)	ND	1 (2.1%)	0 (0%)	1 (2%)	Prolonged stay 3 (7%)	Prolonged hospital stays 15 (35%)
Welp et al.	FS	2 (2.4%)	2 (2.2%)	4 (2.2%)	7 (7.7%)	ND	2 (2.2%)	5 (5.5%)	6 (6.6%)	2.6±6.8	11±9.7
	MS	2 (1.6%)	4 (3.2%)	3 (3.2%)	0 (0%)	ND	2 (1.6%)	2 (1.6%)	11 (8.7%)	2±4.7	10±13.1

Supplementary Table S4.

Author	Intervention	CPB Time	ACC Time	Operation Time	Early Mortality	Midterm Mortality	Late Mortality
Cammertoni et al.	FS	98.6±16.2	72.1±15.6	221.4±33.4	0 (0%)	1 (1.1%)	1 (1.1%)
	MS	109.6±17.5	69.6±14.1	246.6±32.1	1 (1.1%)	2 (2.2%)	2 (2.2%)
Xie et al.	FS	85.5±15.3	56.3±12.6	173.3±41.6	0 (0%)	ND	ND
	MS	90.6±16.5	62.3±14.4	176.3±38.4	0 (0%)	ND	ND
Mikus et al.	FS	68±23.9	56±21.6	ND	8 (4.5%)	ND	ND
	MS	63±20.8	55±19.3	ND	3 (1.1%)	ND	ND
Pisano et al.	FS	92.2±11.8	71.7±15.5	ND	0 (0%)	ND	ND
	MS	102.7±5.9	74.1±5.4	ND	0 (0%)	ND	ND
Welp et al.	FS	108.5±32.7	70±18.9	198.4±47.2	1 (1.09%)	ND	ND
	MS	107.4±24	76.4±18.8	194.1±37.4	1 (0.79%)	ND	ND

Supplementary Table S5.

Study	Selection				Comparability	Outcome			Score
	I	II	III	IV	V	VI	VII	VIII	
Cammertoni et al. 2024	*	*	*	*	**	*	*	--	8
Xie et al. 2022	*	*	*	*	**	*	--	--	7
Mikus et al. 2020	*	*	*	*	**	*	--	--	7
Pisano et al. 2017	*	*	*	*	**	*	--	--	7
Welp et al. 2018	*	*	*	*	**	*	--	--	7

I, Representativeness of the exposed cohort; II, Selection of the non-exposed cohort; III, Ascertainment of exposure; IV, Demonstration that the outcome of interest was not present at start of study; V, Comparability of cohorts on the basis of the design or analysis; VI, Assessment of outcome; VII, Was follow-up long enough for outcomes to occur?; VIII, Adequacy of cohort follow-up.

Supplementary Figure 1.

Need for PPM

Study or Subgroup	Full sternotomy Events	Full sternotomy Total	Ministernotomy Events	Ministernotomy Total	Weight	Odds Ratio M-H, Random, 95% CI	Year
Pisano 2017	2	42	1	42	10.7%	2.05 [0.18, 23.51]	2017
Welp 2018	2	91	2	126	16.2%	1.39 [0.19, 10.08]	2018
Mikus 2020	6	176	7	271	51.8%	1.33 [0.44, 4.03]	2020
Cammertoni 2024	2	91	4	91	21.4%	0.49 [0.09, 2.74]	2024
**Total (95% CI)**		**400**		**530**	**100.0%**	**1.13 [0.51, 2.51]**	
Total events	12		14				

Heterogeneity: Tau² = 0.00; Chi² = 1.27, df = 3 (P = 0.74); I² = 0%
Test for overall effect: Z = 0.31 (P = 0.76)

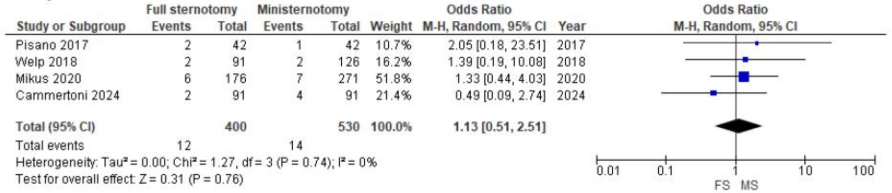

Stroke

Study or Subgroup	Full sternotomy Events	Full sternotomy Total	Ministernotomy Events	Ministernotomy Total	Weight	Odds Ratio M-H, Random, 95% CI	Year
Pisano 2017	0	42	0	42		Not estimable	2017
Welp 2018	5	91	2	126	73.8%	3.60 [0.68, 19.01]	2018
Cammertoni 2024	1	91	1	91	26.2%	1.00 [0.06, 16.23]	2024
**Total (95% CI)**		**224**		**259**	**100.0%**	**2.57 [0.62, 10.74]**	
Total events	6		3				

Heterogeneity: Tau² = 0.00; Chi² = 0.60, df = 1 (P = 0.44); I² = 0%
Test for overall effect: Z = 1.30 (P = 0.19)

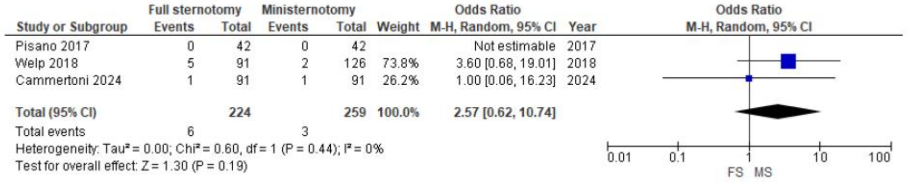

Re-Exploration

Study or Subgroup	Full sternotomy Events	Full sternotomy Total	Ministernotomy Events	Ministernotomy Total	Weight	Odds Ratio M-H, Random, 95% CI	Year
Pisano 2017	0	42	0	42		Not estimable	2017
Welp 2018	4	91	3	126	43.3%	1.89 [0.41, 8.64]	2018
Xie 2022	0	60	1	60	9.7%	0.33 [0.01, 8.21]	2022
Cammertoni 2024	5	91	3	91	47.0%	1.71 [0.40, 7.36]	2024
**Total (95% CI)**		**284**		**319**	**100.0%**	**1.52 [0.56, 4.14]**	
Total events	9		7				

Heterogeneity: Tau² = 0.00; Chi² = 0.98, df = 2 (P = 0.61); I² = 0%
Test for overall effect: Z = 0.82 (P = 0.41)

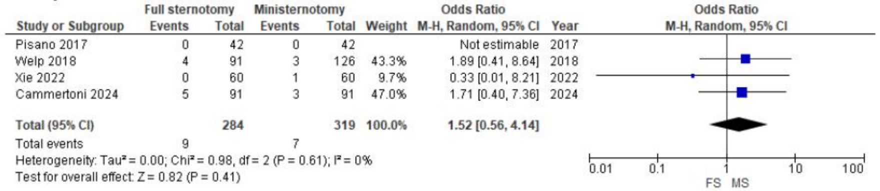

Renal Failure

Study or Subgroup	Full sternotomy Events	Full sternotomy Total	Ministernotomy Events	Ministernotomy Total	Weight	Odds Ratio M-H, Random, 95% CI	Year
Pisano 2017	1	42	0	42	7.2%	3.07 [0.12, 77.59]	2017
Welp 2018	2	91	2	126	19.2%	1.39 [0.19, 10.08]	2018
Mikus 2020	8	176	4	271	51.0%	3.18 [0.94, 10.72]	2020
Xie 2022	2	60	3	60	22.6%	0.66 [0.11, 4.07]	2022
Cammertoni 2024	0	91	0	91		Not estimable	2024
**Total (95% CI)**		**460**		**590**	**100.0%**	**1.89 [0.80, 4.51]**	
Total events	13		9				

Heterogeneity: Tau² = 0.00; Chi² = 2.17, df = 3 (P = 0.54); I² = 0%
Test for overall effect: Z = 1.44 (P = 0.15)

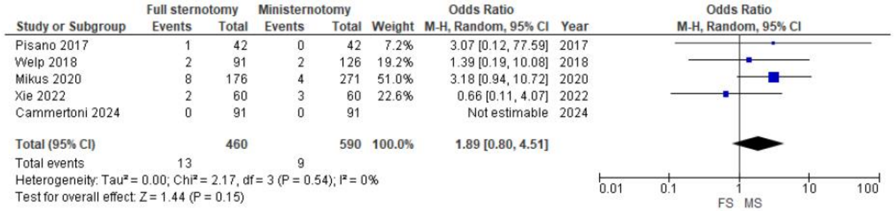

NOAF

Study or Subgroup	Full sternotomy Events	Full sternotomy Total	Ministernotomy Events	Ministernotomy Total	Weight	Odds Ratio M-H, Random, 95% CI	Year
Pisano 2017	10	42	6	42	8.1%	1.88 [0.61, 5.74]	2017
Welp 2018	2	91	4	126	3.4%	0.69 [0.12, 3.82]	2018
Mikus 2020	69	176	117	271	67.8%	0.85 [0.58, 1.25]	2020
Cammertoni 2024	23	91	18	91	20.7%	1.37 [0.68, 2.76]	2024
**Total (95% CI)**		**400**		**530**	**100.0%**	**0.99 [0.72, 1.36]**	
Total events	104		145				

Heterogeneity: Tau² = 0.00; Chi² = 2.87, df = 3 (P = 0.41); I² = 0%
Test for overall effect: Z = 0.05 (P = 0.96)

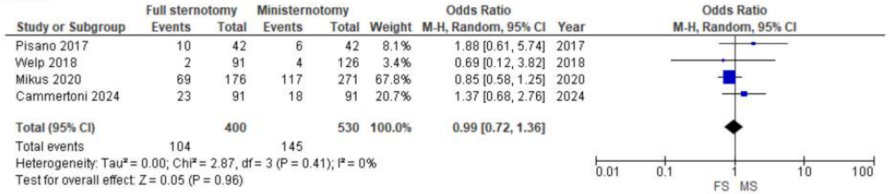

Wound dehiscence

Study or Subgroup	Full sternotomy Events	Full sternotomy Total	Ministernotomy Events	Ministernotomy Total	Weight	Odds Ratio M-H, Random, 95% CI	Year
Pisano 2017	0	42	1	42	5.5%	0.33 [0.01, 8.22]	2017
Welp 2018	6	91	11	126	49.8%	0.74 [0.26, 2.07]	2018
Mikus 2020	4	176	2	271	19.1%	3.13 [0.57, 17.26]	2020
Xie 2022	0	60	1	60	5.5%	0.33 [0.01, 8.21]	2022
Cammertoni 2024	5	91	2	91	20.1%	2.59 [0.49, 13.69]	2024
**Total (95% CI)**		**460**		**590**	**100.0%**	**1.14 [0.54, 2.44]**	
Total events	15		17				

Heterogeneity: Tau² = 0.02; Chi² = 4.10, df = 4 (P = 0.39); I² = 3%
Test for overall effect: Z = 0.35 (P = 0.73)

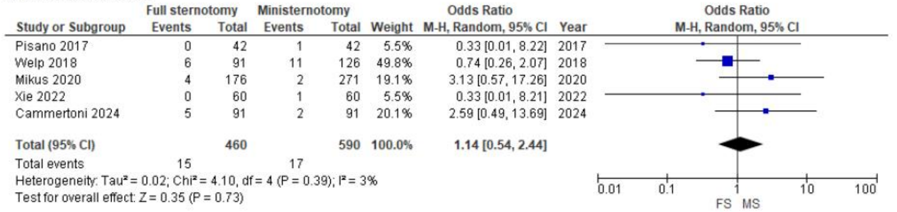

Supplement: Supplementary Table S1 — Multivariate logistic regression analysis. [file Datasheet1.pdf]
